# Laparoscopic versus open minor liver resections of colorectal cancer liver metastases in a state at war: A prospective cohort study

**DOI:** 10.1016/j.sopen.2026.03.006

**Published:** 2026-03-20

**Authors:** Anton Burlaka, Serhii Zemskov, Veronika Rozhkova, Vitaliy Zvirych, Mykhailo Dupyk, Andrii Beznosenko, Volodymyr Bezverkhnyi, Volodymyr Skyba

**Affiliations:** a National cancer institute, Division of thoraco-abdominal oncology, Kyiv, Ukraine; b Bogomolets National Medical University, Kyiv, Ukraine; c Military Hospital, Irpin, Ukraine

**Keywords:** Colorectal cancer, Liver metastases, Laparoscopic liver resection, Laparoscopic surgery costs, State hospital

## Abstract

**Introduction:**

In 2017 the laparoscopic left lateral sectionectomy and minor resections of the anterior liver segments were considered the standard of practice worldwide. However, in 2023 only 5% of liver resections were conducted laparoscopically within the Ukrainian specialized centers.

**Aim:**

To investigate whether the laparoscopic minor liver resections (LLR) have better financial, surgical and long-term oncological outcomes compared to open liver resections (OLR) in the two state specialized hospitals in Ukraine**.**

**Methods:**

Between January 2022 and June 2025, 205 patients with colorectal liver metastases (CLM) received either minor OLR or LLR. Primary end-points were the 30-day morbidity, surgical outcomes. Secondary endpoints were hospital costs and the oncological outcomes.

**Results:**

There was a difference in postoperative morbidity between the groups in favor of the LLR – 3.8% (*n* = 4) vs 11.7% (*n* = 12) in OLR (*p* = 0.04). 1.9% (*n* = 2) in the OLR group had major complications. No mortality was registered in either group. The final sum of costs for treatment of 103 patients in LLR vs 102 patients in OLR was 3.8 M UAH vs 3.5 M UAH, respectively (*p* = 0.82). The 2-year recurrence-free survival (RFS) was 44% vs 54%, and 19.6 months vs 24.0 months respectively for OLR and LLR groups (*p* = 0.62)**.**

**Conclusions:**

The laparoscopic minor liver resections in patients with colorectal cancer liver metastases in state specialized hospitals reduced the recovery period and the postoperative morbidity level with similar oncological outcomes and costs**.**

## Highlights

• Laparoscopic liver resections are increasingly implemented in Ukraine despite economic crisis
• MILS allows to achieve better surgical results without major financial toxicity
• Minor liver resections can be safely performed laparoscopically in countries with transitioning economy
• Minimizing the use of disposable equipment, application of reusable supplies might improve the cost effectiveness of MILS

## Background

Colorectal cancer (CRC) is the fourth most common malignancy in Ukraine with 15,022 new cases diagnosed annually [1]. Synchronous liver metastases (LM) are found in 25% of cases, whereas metachronous manifestation of metastatic disease is registered in up to 60% of patients [2]. Over the last decades surgical treatment of LM demonstrated the most favorable oncological results, with achievement of R0-resection margin remaining one of the most important prognostic factors. The minimally invasive surgery has been increasingly implemented in surgical management of both primary and secondary liver lesions, and has demonstrated multiple advantages [3]. However Ukrainian surgical centers predominantly rely on open liver surgery – in 2020 only 5% of liver resections were performed laparoscopically, while worldwide the laparoscopic left lateral sectionectomy and minor resections of the anterior liver segments were considered the standard practice [4], [5], [6].After the series of successful studies, many hospitals actively implemented the laparoscopic liver resections (LLR) in the routine practice [7], [8], [9]. In 2023 the population-based study of minimally invasive liver surgery (MILS) outcomes from Norway centers, showed that the 37.8% of cases were performed laparoscopically [10]. This study confirmed the benefits of LLR in short-term results, while survival was comparable to open liver surgery. Selective randomized trials demonstrated cost-effectiveness of LLR compared to open surgery with up to 67% probability [11], [12]. However all these studies relied on data from the economically developed countries, so the question of LLR efficiency in countries experiencing economic crisis remains open.

After the 2020 MILS was adopted in the National Cancer Institute clinic in Kyiv and in 2022 this study was initiated. Since 2023 the healthcare system in Ukraine has been under complex reconstruction, with war remaining a serious threat for cancer patients in state hospitals [13], [14]. Also, the new system of public reimbursement was implemented which set the limits to financially toxic treatment strategies. Thus, the aim of this study was to investigate if the LLR have better financial, surgical and long-term oncological outcomes compared to open liver resections (OLR) in two state specialized hospitals in Ukraine.

## Methods

**Fig. 1:**
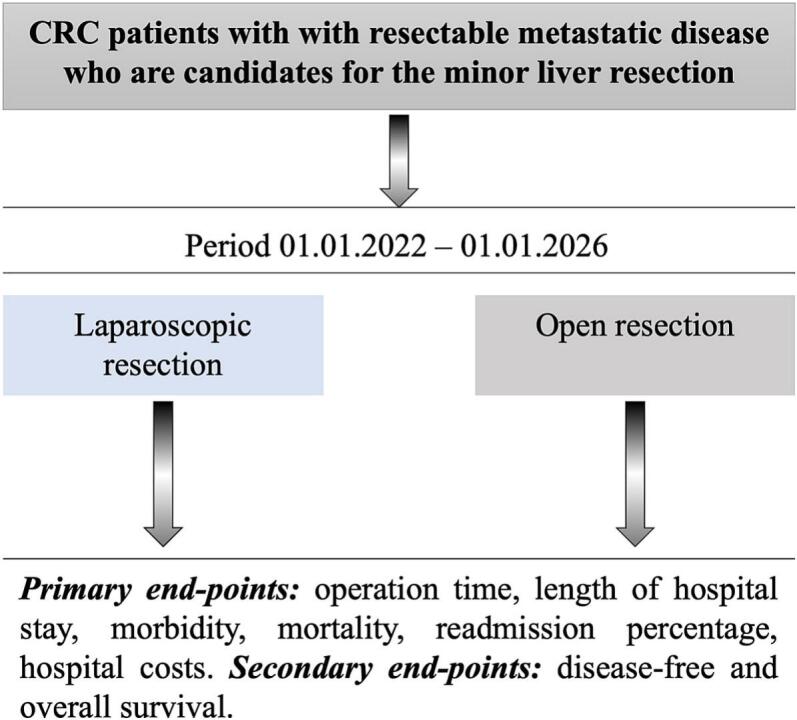
Schematic representation of the study design.

This is a prospective analysis of the results of 205 patients with colorectal cancer liver metastases who received either minor OLR or LLR between January 2022 and June 2025 in two specialized hospitals in Kyiv, Ukraine – National Cancer Institute and Bogomolets University Hospital (Fig. 1) [15].

Inclusion criteria were: CRC patients with LM, candidates for minor liver resections (≤ 2 liver segments), aged 18—82 years, ASA physical status I-III, and ECOG 0–1 who signed an informed consent to participate in the study. We excluded patients who were unable/unwilling to sign an informed consent, patients who previously underwent liver ablations, and patients with unresectable extrahepatic disease.

Surgical techniques in cohorts included parenchyma-sparing liver resection. All the surgical procedures were performed by HPB-trained surgeons experienced in open and laparoscopic liver surgery (≥50 resections). Primary endpoints were the surgical outcomes, and 30-days morbidity rate the secondary endpoints were hospital costs and the long-term outcomes. IWATE difficulty scoring system was used to assess the difficulty of laparoscopic liver resections [16].

All liver resection technique included crush-clamping or a cavitron ultrasonic surgical aspirator with a resection margin size of at least 1 mm. When feasible, tactics of “vascular detachment” were be used. The ischemia technique included the classical Pringle maneuver (20 min ischemia, 5 min reperfusion). All parenchyma-sparing LRs were accompanied by intraoperative ultrasound navigation.

Costs were estimated based on medical files records, with a focus on a cost of the surgery itself, the consumables, and a hospital stay. The procurement as well as purchase prices were taken from publicly available sources as well as from the internal documents. Costs were calculated in Ukrainian hryvnia (UAH) on a present-value basis.

**Statistics.** The normality of the distribution was analyzed with the Shapiro-Wilk test. Summary statistics were presented as whole numbers and percentages for categorical variables, medians with interquartile ranges for continuous variables, and means with deviations from the standard error for interval data with a normal distribution. Survival rates were assessed using the Kaplan-Meier method and calculated from the day of liver surgery. Tukey's multiple comparisons test was used for non-parametric multiple comparisons in Clavien-Dindo groups. A multiple linear regression method was used to assess the association of treatment costs and clinical or pathological factors. The data were analyzed using the statistical package Prizm 10.5.

## Results

### Study population

This study included 205 CRC patients with LM, 103 of who underwent LLR. The groups' distribution by gender, age and BMI was similar (Table 1). Left-sided primary tumors were diagnosed in 86.4% (*n* = 89) and 66.6% (*n* = 83) of patients in the LLR and OLR groups respectively. There was no difference in the diameter of metastatic lesions (3.4 cm vs 3.1 cm, *p* = 0.2), nor in the average number of the resected metastases (1.5 and 1.6, *p* = 0.8 for OLR and LLR respectively). Metastatic liver lesions were synchronous in 41.7% (*n* = 43) of LLR and in 30.4% (*n* = 31) of OLR groups (*p* = 0.09). The IWATE difficulty score of laparoscopic surgery was 2.4 ± 0.2. In 16.5% (*n* = 17) of the LLR and in 23.5% (*n* = 24) of the OLR cases simultaneous surgery of the liver and primary tumor was performed (*p* = 0.2). Liver re-resections were performed in 5.8% (*n* = 6) and 8.8% (*n* = 9) of cases in laparoscopic and open surgical approach respectively (*p* = 0.4). The number of the chemotherapy cycles that the patients received was similar (Table 1).

**Table 1:** Patient demographic and clinical results.

	LLR (103)	OLR (102)	*P*-value
F/M	47 (45.6) / 55 (54.4)	44 (43.2) / 57 (56.8)	0.4
Age	59.9 (33–72)	61.3 (38–76)	0.8
BMI	27.65 (2.94)	28.21 (2.78)	0.3
Size of largest LM, cm	3.1 (2–4)	3.4 (1–6)	0.2
Number of resected tumors	1.5 ± 0.2	1.6 ± 0.2	0.8
Synchronous status of liver metastases	43 (41.7)	31 (30.4)	0.09
Left-sided primary tumor localization	89 (86.4)	83 (66.6)	0.3
IWATE difficulty score	2.4 ± 0.2	–	
Simultaneous surgery^a^	17 (16.5)	24 (23.5)	0.2
Liver re-resection	6 (5.8)	9 (8.8)	0.4
Chemotherapy (courses)	8.9 ± 0.4	9.7 ± 0.4	0.2
30-day morbidity:	4 (3.8)	12 (11.7)	0.04
• Bile leakage	–	2 (1.9)	0.2
• Intra-abdominal infection	–	3 (2.9)	0.08
• Surgical site infection	1 (0.9)	6 (5.8)	0.054
• Pneumonia/pleuritis	3 (2.9)	1 (0.9)	0.3
• Cardiac complication	–	1 (0.9)	0.3
Major morbidity	–	2 (1.9)	0.2
Mortality	–	–	
ICU admission on 1 POD	3 (2.9)	26 (25.5)	<0.01
*Re*-admissions	–	2 (1.9)	0.2
Average stay (Mean ± SD)	3.3 ± 0.8	8.9 ± 2.6	<0.01
Blood loss, ml	336.4 ± 165	343.6 ± 167	0.6
Surgery duration, min	413 ± 202	387.5 ± 189	0.3

a In the group of simultaneous resections, only the cost of liver surgery was analyzed.

### Surgical outcomes

The morbidity level in the LLR vs OLR was 3.8% (n = 4) vs 11.7% (*n* = 12) (*p* = 0.04). Major morbidity was observed exclusively in 1.9% (*n* = 2) of patients in the OLR group. In OLR cohort, one patient required percutaneous drainage of the biloma and one patient was diagnosed with deep wound infection and eventration, which required antibiotic therapy and prolonged recovery with subsequent formation of a ventral hernia. Minor surgical site infection occurred in 0.9% (*n* = 1) and 5.8% (*n* = 6) of cases in LLR and OLR respectively (*p* = 0.54). Also, we registered bile leakage in 0 vs 1.9%, intra-abdominal infection in 0 vs 2.9%, non-specific pleuritis or pneumonia in 2.9% vs 0.9% and atrial fibrillation in 0 vs 0.9% of patients in LLR and OLR respectively. No mortality was registered in both groups.

MILS required from 4 to 5 trocars, depending on liver segment, vascular invasion, parenchyma quality, etc. Supine position was the best option for left lobe or S5 lesions, while posterior and doom segments demanded Jack-knife or Semi-prone positions (Table 2). Intermitted intracorporal Pringle maneuver was applied as in-flow control with ischemia duration range of 10–27 min. Open surgical approach included supraumbilical midline or J-shaped to the right laparotomy in 40.1% and 59.9% respectively. In the open surgery group, all patients received epidural analgesia, whereas in the LLR group, we used TAP blocks and QL blocks.

**Table 2:** Surgical and technical characteristics of LLRs.

Liver segments	Trocars amount	Patient position	Vessel Control	Mean warm ischemia duration, min
S1	4	Supine	–	–
S2–3	4	Supine	–	–
S4	5	Supine	PM with HV outflow control	23
S5	5	Supine	PM	27
S6	4	Semi-prone	PM	10
S7	5	Jack-knife with split legs or Semi-prone	PM with HV outflow control	20
S8	5	Semi-prone	PM with HV outflow control	26

**Fig. 2:**
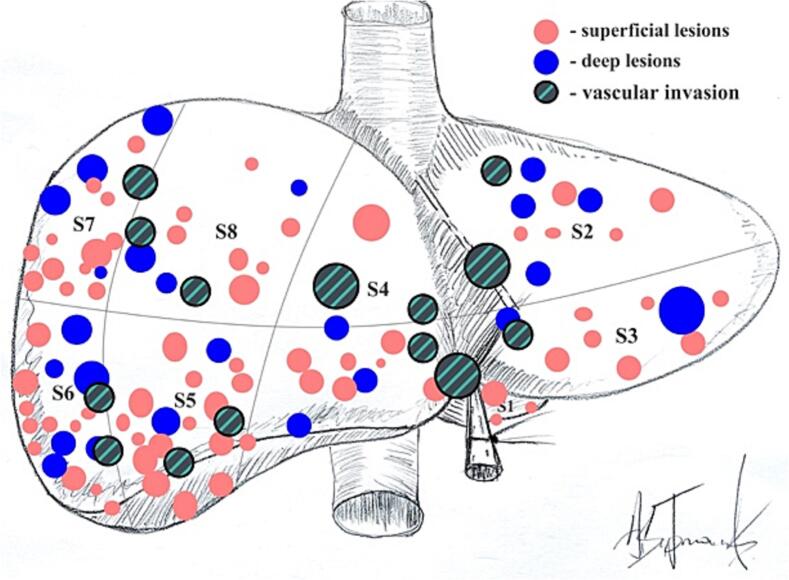
Map of resected LM in LLR group. Superficial lesions - LM that could be identified without intraoperative ultrasound (IOS), deep lesion – required IOS navigation, vascular invasion - radiologically or intraoperatively confirmed intrahepatic 1st-3rd order veins of glissonean pedicles invasion that required subsequent vessels ligation.

Approximately 60% of LM were localized in the left lateral section and in the inferior segments (S4b, S5, S6), while posterior-superior segments (S4a, S7, S8) were resected in 40% of the LLR group. During laparoscopic surgery, 23.3% (*n* = 24) of patients required intraoperative ultrasound LM detection (deep lesions), vascular invasion detected in 13.6% (*n* = 14) of LLR cases (Fig. 2). For simultaneous surgery in OLR group, total midline laparotomy was applied in most cases. The trocar placement was adopted for simultaneous LLR with taking into account triangulation and the surgeon's experience [17].

There was no difference in blood loss and surgery duration between the groups (Table 1). Enhanced monitoring in the intensive care unit on postoperative day 1 was applied in 2.9% (*n* = 3) and 25.5% (*n* = 26) cases of LLR and OLR groups respectively (*p* < 0.01). Hospital readmission for the surgical complications management was 0 vs 1.9% (n = 2) in the LLR and OLR cohorts respectively (*p* = 0.2). The duration of hospital stay in the OLR group was significantly longer compared to laparoscopic resections: 8.9 ± 2.6 vs 3.3 ± 0.8 (p < 0.01).

### Costs

**Fig. 3:**
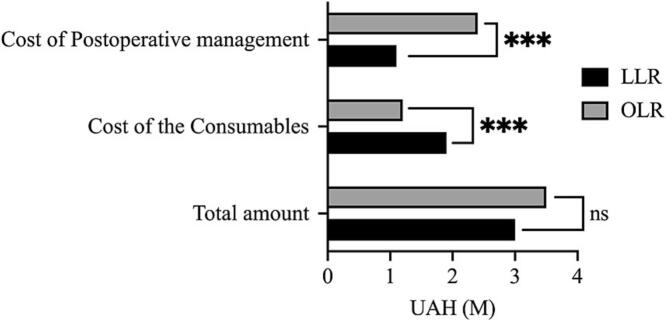
Scatter plot, graphical comparing of postoperative management and surgical materials costs in LLR and OLR groups; *** - *p* < 0.01.

The largest share of expenses in LLR were disposable laparoscopic trocars – 559,109 UAH, vessel sealing devices – 1,151,400 UAH and vascular staplers – 75,000 UAH (Table 3). Also, the disposable bipolar clamps were used for LLR, amounting to 25,333 UAH. In the OLR group the most expensive consumables were titanium clips – 140,679 UAH and vessel sealing devices – 878,700 UAH. The total cost of consumables in the LLR group was higher comparing to the OLR group, amounting up to 1,974,750 UAH and 1,157,535 UAH, respectively (*p* < 0.01) (Fig. 3). The final sum of costs for treatment of 103 patients in LLR vs 102 patients in OLR was 3.8 M UAH vs 3.5 M UAH, respectively (*p* = 0.82).

**Table 3:** Estimation of basic expenses in comparison groups.

	LLR	OLR	P-value
Cost of the consumables:			
Laparoscopic trocars	559,109	–	<0.01
Vascular staplers	75,000	21,000	0.004
Manual titanium surgery clips	53,766	140,679	<0.01
Hem-o-lok type clips	39,273	–	<0.01
Ultrasonic surgical aspirator (CUSA)	60,000	30,000	0.18
Bipolar electrosurgery (single use)	25,333	–	<0.01
Vessel sealing devices (single use)	1,151,400	878,700	0.42
Manual sutures	3000	72,450	<0.01
Other	7869	14,706	
Total amount of the consumables	**1,974,750**	**1,157,535**	**<0.01**

Cost of postoperative management:			
ICU management	50,000	400,000	<0.01
*Re*-admissions management	–	60,000	<0.01
PO morbidity management	85,000	355,000	<0.01
In-hospital stay	902,304	1,536,336	<0.01
Total amount of the postoperative management	**1,037,304**	**2,351,336**	**<0.01**
Overall amount	**3,841,500**	**3,562,871**	**0.82**

Expenses for post-surgical monitoring and support within the intensive care unit were higher for OLR groups: more than 400,000 UAH comparing to 50,000 UAH in LLR (*p* < 0.01) (Table 3). Price of re-admissions management due to complications after discharge was 0 vs 60,000 UAH for LLR and OLR respectively (*p* < 0.01). The costs of postoperative management for LLR amounted to 1,037,304 UAH and 2,351,336 in OLR group (p < 0.01).

**Fig. 4:**
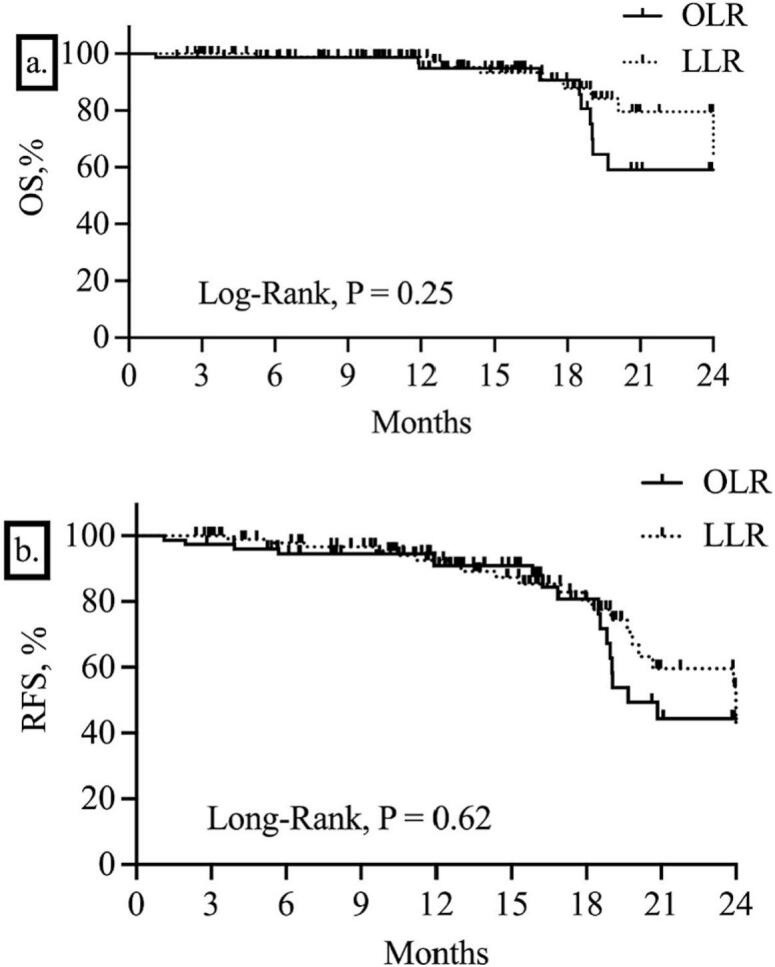
OS (a) and RFS (b) curves (Kaplan-Meier) for patients with CRC and LM who underwent LLR and OLR.

The median follow-up period was 13.6 months, and 2-years OS was 59% and 69% in OLR and LLR groups respectively with undefined median survival (*p* = 0.25) (Fig. 3a.). Disease recurrence registered in 18 and 21 patients in the OLR and LLR groups. 2-years recurrence-free (RFS) was 44% vs 54% and median of RFS was 19.6 months vs 24.0 months respectively for OLR and LLR (*p* = 0.62) (Fig. 4b).

According to the regression analysis, a significant impact on the cost of treatment was found for hospital stays longer than 3.3 days, postoperative complications, postoperative ICU monitoring and metastasis localization in the posterio-superior segments (Table 4).

**Table 4:** Multivariate analysis.

Variable	95% CI (profile likelihood)	|t|	*P* value	R2 with other variables
Intercept	12,335 to 27,645	5.15	0.00	
Laparosopic surgery	−4850 to 2793	0.53	0.60	0.49
In-hospital stay ≥3.3 days	**−3687 to −1263**	**4.03**	**0.00**	**0.59**
PO morbidity	**5273 to 11,870**	**5.13**	**0.00**	**0.26**
*Re*-admissions	−19,396 to 10,366	0.60	0.55	0.14
ICU monitoring	**11,388 to 22,453**	**6.03**	**0.00**	**0.50**
Lesions number (≥3)	−19,396 to 10,366	0.21	0.72	0.04
Posterio-superior segments	**3891 to 14,980**	**3.36**	**0.00**	**0.49**

## Discussion

Open incisions in the context of liver surgery are highly traumatic and associated with a prolonged postoperative hospital stay up to 12 postoperative days and wound site infections rate of up to 13% [18], [19], [20]. This applies especially to patients with non-healthy liver, when the organ is enlarged, and its mobilization requires extended incision which carries risks of morbidity and affects patient's quality of life [21]. Over the last two decades, there has been a progressive shift towards the increased use of MILS with three published international consensus statements [6], [22]. Most of the randomized trials and meta-analysis consider minor LLR as safe and effective approach for primary and secondary liver malignancies [23], [24], [25]. The principal counter arguments in debates regarding the use of a laparoscopic approach in liver surgery relate to financial considerations. Costs of the laparoscopic liver surgery vary depending on the type of liver resection and country-specific reimbursement system [26]. Cost drivers such as postoperative complications and the use of expensive energy devices/staplers remain the main contributors to increased overall expenses. Few large retrospective studies have compared the costs of major open and laparoscopic liver resections, and they demonstrated significantly higher intraoperative costs for MILS mainly due to the laparoscopic instruments [27], [28]. However, some hospitals have experience of costs reducing in minor LLR when the surgical team reached expert level [11]. Also, it is important to consider that MILS is technically more complex and more demanding in anatomical landmarks, navigation, R0 margins achievement, bleeding control, etc. [29]. Mastering this approach requires passing the learning curve with up to 50 minor liver resections which can be improved with specialized fellowship programs [30]. Whereas advanced- and expert-difficult procedures according to IWATE criteria score should be performed in highly specialized centers by expert team. The high-priced radiological navigation systems and specialized surgical equipment for precise laparoscopic liver surgery are rarely encountered in state hospitals of our country.

Unfortunately, the course of this study has been influenced by war and healthcare reforms in Ukraine [31]. However, the obtained results allowed to improve the quality of surgical and oncological care for CRC patients and confirmed the ‘global experience’. Also, the recovery time without the intensive care support and the postoperative morbidity were minimized in two public specialized hospitals. It became clear that to major financial toxicity contributes the application of disposable laparoscopic surgical materials. However, the final cost of treatment in the groups didn't demonstrate a statistical difference. We believe that minimizing the use of disposable equipment, application of reusable supplies and optimization of transection techniques will help us to improve the cost effectiveness of LLR.

Finally, the importance of experience in open surgery should not be underestimated, as experts in open liver resections often serve as pace-setters in the adoption of laparoscopic liver resection. Moreover, the introduction of minimally invasive liver surgery in both participating centers accelerated the evolution of surgical techniques among operating teams in both open and laparoscopic surgery.

In conclusion, laparoscopic minor liver resections in patients with colorectal cancer liver metastases in state specialized hospitals reduced the recovery period and the postoperative morbidity level with similar oncological outcomes and costs.

### Limitations

Laparoscopic surgery cost is not just limited to surgical expenses or immediate results. When comparing open surgery with laparoscopic, we forget to consider the cost of personnel training (nurses, surgeons, radiologists and technicians) as well as the cost of additional equipment that makes it possible to perform this complex surgery through a minimally invasive approach. Other limitations of this study are relatively short follow-up and small sample size. Furthermore, retrospective studies are prone to selection bias.

## CRediT authorship contribution statement

**Anton Burlaka:** Methodology, Formal analysis, Conceptualization. **Serhii Zemskov:** Supervision, Project administration, Data curation. **Veronika Rozhkova:** Writing – review & editing, Writing – original draft, Validation. **Vitaliy Zvirych:** Validation, Supervision, Project administration. **Mykhailo Dupyk:** Methodology, Investigation, Data curation. **Andrii Beznosenko:** Validation, Project administration, Investigation. **Volodymyr Bezverkhnyi:** Investigation, Formal analysis, Conceptualization. **Volodymyr Skyba:** Resources, Project administration, Conceptualization.

## Ethical approval and informed consent

Informed consent was obtained from all individual participants included in the study in compliance with the Helsinki Declaration. The Ethics Service committee of National Cancer Institute (Project ID 211/9 on 11 January 2022) approved this study.

## Funding source

The Ministry of Health of Ukraine (no. 0121U110087).

## Declaration of competing interest

None declared.

## Data Availability

The datasets used and/or analyzed during the current study are available from the corresponding author on reasonable request.
